# Cultivable Anaerobic Microbiota of Infected Root Canals

**DOI:** 10.1155/2012/609689

**Published:** 2012-04-03

**Authors:** Takuichi Sato, Keiko Yamaki, Naoko Ishida, Kazuhiro Hashimoto, Yasuhisa Takeuchi, Megumi Shoji, Emika Sato, Junko Matsuyama, Hidetoshi Shimauchi, Nobuhiro Takahashi

**Affiliations:** ^1^Division of Oral Ecology and Biochemistry, Tohoku University Graduate School of Dentistry, Sendai 980-8575, Japan; ^2^Division of Periodontology and Endodontology, Tohoku University Graduate School of Dentistry, Sendai 980-8575, Japan; ^3^Division of Advanced Prosthetic Dentistry, Tohoku University Graduate School of Dentistry, Sendai 980-8575, Japan; ^4^Division of Pediatric Dentistry, Niigata University Graduate School of Medical and Dental Sciences, Niigata 951-8514, Japan

## Abstract

*Objective*. Periapical periodontitis is an infectious and inflammatory disease of the periapical tissues caused by oral bacteria invading the root canal. In the present study, profiling of the microbiota in infected root canals was performed using anaerobic culture and molecular biological techniques for bacterial identification. *Methods*. Informed consent was obtained from all subjects (age ranges, 34–71 years). Nine infected root canals with periapical lesions from 7 subjects were included. Samples from infected root canals were collected, followed by anaerobic culture on CDC blood agar plates. After 7 days, colony forming units (CFU) were counted and isolated bacteria were identified by 16S rRNA gene sequencing. *Results*. The mean bacterial count (CFU) in root canals was (0.5 ± 1.1) × 10^6^ (range 8.0 × 10^1^–3.1 × 10^6^), and anaerobic bacteria were predominant (89.8%). The predominant isolates were *Olsenella* (25.4%), *Mogibacterium* (17.7%), *Pseudoramibacter* (17.7%), *Propionibacterium* (11.9%) and *Parvimonas* (5.9%). *Conclusion*. The combination of anaerobic culture and molecular biological techniques makes it possible to analyze rapidly the microbiota in infected root canals. The overwhelming majority of the isolates from infected root canals were found to be anaerobic bacteria, suggesting that the environment in root canals is anaerobic and therefore support the growth of anaerobes.

## 1. Introduction

Periapical periodontitis is an infectious and inflammatory disease of the periapical tissues caused by oral bacteria invading the root canal, and thus resulting in the formation of endodontic lesions. The microbiota in infected root canals has been reported to consist of anaerobic bacteria, by the adoption of various improved anaerobic culturing techniques, such as an anaerobic glove box [1–3]. However, identification of bacterial species by utilizing only these techniques is time consuming and labor intensive. In recent years, molecular biological methods such as random cloning and 16S rRNA sequence analysis have been introduced in order to profile the microbiota [4, 5]. However, live and dead bacterial cells in the microbiota could not be differentiated by utilizing only the molecular biological methods, and in consequence, the pathogenicity of live bacterial cells in the endodontic lesions remains to be fully determined.

 Therefore, in the present study, we applied molecular biological techniques, that is, restriction fragment length polymorphism analysis of PCR-amplified 16S ribosomal RNA genes (PCR-RFLP) and sequencing, to bacterial colonies after anaerobic culture, for bacterial identification, in order to clarify the composition of live bacterial cells of the microbiota in infected root canals.

## 2. Materials and Methods

### 2.1. Subjects

Subjects with periapical periodontitis (five females and two males; age, 34–71 years), who were attending the Clinical Division of Endodontology, Tohoku University Hospital, Sendai, Japan, were randomly selected for this study. Periapical periodontitis was diagnosed based on clinical features, that is, putrefactive smell, spontaneous pain, sensitivity (tenderness) to percussion/occlusion, pus discharge, swellings and fistula, and radiographical findings. Selected teeth had clinically no obvious margin leakages, had enough coronal structure for adequate isolation with a rubber dam, and were free of periodontal pockets deeper than 4 mm. Based on history, all subjects were medically healthy and received no antibiotics for the 3 months before sampling. Informed consent was obtained from all subjects, and this study was approved by the Research Ethics Committee of Tohoku University Graduate School of Dentistry, Sendai, Japan.

### 2.2. Sampling and DNA Extraction

Each tooth was isolated with a rubber dam, and the operative field was disinfected with both iodine glycerin dental disinfectants Showa (Showa Yakuhin Kako, Tokyo, Japan) and 70% ethanol. Coronal access cavity was prepared with a sterilized high-speed bur under irrigation with sterile saline solution. When the pulp chamber was exposed, a sterile no. 15 K-file (GC, Tokyo, Japan) was introduced and the canal length was determined using an apex locator (Root ZX, Morita, Japan). The dentin sample was collected from an apical canal by filing intensively with a sterile K-file of the canal size. After the sampling, cleaning and shaping of the root canal was carried out with sterile K-files (from #15 to #55, GC, Tokyo, Japan). An intracanal medicament, that is, calcium hydroxide paste (UltraCal XS, Ultradent Products Inc., South Jordan, UT, USA) was applied, and the coronal access cavity was sealed with a temporary cement (Lumicon; Heraeus Kulzer Japan, Tokyo, Japan).

 Each file cut off by a sterilized wire cutter was immediately transferred to an anaerobic glove box (Hirasawa, Tokyo, Japan) containing 80% N_2_, 10% H_2_, and 10% CO_2_. While in the box, each sample was suspended in 1.0 mL of sterilized 40 mM potassium phosphate buffer (pH 7.0), and, after vortexing, the suspension was dispersed with a Teflon homogenizer. Serial 10-fold dilutions (0.1 mL each) were spread onto the surface of CDC anaerobe 5% sheep blood agar (BD, Franklin Lakes, NJ, USA) plates (duplicate) and incubated in the anaerobic glove box at 37°C for 7 days. After the incubation, colony-forming units (CFUs) were counted, and all colonies from suitably diluted plates having <100 colonies (mean 13.1; range 8–31 colonies) were subcultured.

### 2.3. DNA Extraction and Identification by DNA Sequence Analysis

Genomic DNA was extracted from each single colony with the InstaGene Matrix Kit (Bio-Rad Laboratories, Richmond, CA, USA) according to the manufacturer's instructions.

 The 16S rRNA gene sequences were amplified by PCR using universal primers 27F and 1492R and *Taq* DNA polymerase (Hot Star *Taq* Master Mix, Qiagen GmbH, Hilden, Germany) according to the manufacturer's instructions. Primer sequences were: 27F; 5′-AGA GTT TGA TCM TGG CTC AG-3′ and 1492R, 5′-TAC GGY TAC CTT GTT ACG ACT T-3′ [6, 7]. Amplification proceeded using a PCR Thermal Cycler MP (TaKaRa Biomedicals, Ohtsu, Shiga, Japan) programmed as follow: 15 min at 95°C for initial heat activation and 30 cycles of 1 min at 94°C for denaturation, 1 min at 55°C for annealing, 1.5 min at 72°C for extension, and 10 min at 72°C for final extension. PCR products were separated on 1% agarose gels (High Strength Analytical Grade Agarose, Bio-Rad Laboratories) in Tris-borate EDTA buffer (100 mM Tris, 90 mM borate, 1 mM EDTA, pH 8.4), stained with ethidium bromide and photographed under UV light, and their sizes (ca 1466 bp) were confirmed comparing with the molecular size marker (a 100 bp DNA Ladder, Invitrogen, Carlsbad, CA, USA).

 The 16S rRNA genes were individually digested with *Hpa*II (FastDigest, Fermentas, Cosmo Bio, Tokyo, Japan) according to the manufacturer's instructions. Digestion products were separated on 2% agarose gels as described above.

 Isolates were identified tentatively according to RFLP analysis [8–13] as well as morphological data, that is, colony appearances and Gram staining. Then, representative isolates were conclusively identified by sequence analysis as described below, and there were no exceptions among the same RFLP groups. The PCR products were purified with illustra GFX PCR DNA and Gel Band Purification Kit (GE Healthcare, Buckinghamshire, UK) and then sequenced at Fasmac (Atsugi, Kanagawa, Japan) using the BigDye Terminator Cycle Sequencing Kit and an automated DNA sequencer (PRISM-3100, Applied Biosystem Japan, Tokyo, Japan). Primer 1492R was used to sequence (at least 900 bp), and the partial 16S rRNA gene sequences were then compared with those from the GenBank database using the BLAST search program through the website of the National Center for Biotechnology Information. Bacterial species were determined by percent sequence similarity (>97%).

## 3. Results

The mean bacterial count (CFU) in root canals was (0.5 ± 1.1) × 10^6^ (range 8.0 × 10^1^–3.1 × 10^6^) (Table 1).

 The predominant gerera were *Olsenella* (25.4%), *Mogibacterium* (17.7%), *Pseudoramibacter* (17.7%), *Propionibacterium* (11.9%), and *Parvimonas* (5.9%), thus indicating that anaerobic bacteria were totally predominant in infected root canals (89.8%) (Table 2). At the species level, *Mogibacterium timidum* (17.7%), *Pseudoramibacter alactolyticus* (17.7%), *Olsenella profusa* (14.4%) and *Parvimonas micra* (5.9%) were predominant (Table 2).

## 4. Discussion

In the present study, the vast majority of the isolates from infected root canal dentin were found to be anaerobic bacteria (Table 2). The result suggests that the environment of infected root canals is anaerobic and therefore supports the growth of anaerobic bacteria. The finding is in accordance with the previous studies on samples taken from oral cavities, when similar anaerobic incubation procedures were used [2, 3, 14–17].

 The anaerobic strains, belonging to *Olsenella*, *Mogibacterium*, *Pseudoramibacter*, *Propionibacterium, *and *Parvimonas* consisted of the majority of isolates from infected root canals in the present study (Table 2), in agreement with the previous studies [1, 2]. In addition to *Pseudoramibacter *and *Parvimonas*, in those studies [1, 2], *Fusobacterium* was also found among the predominant genera of isolates. These findings suggest that some anaerobic bacteria are common in infected root canals, and that these bacteria may contribute to play some etiological roles for endodontic infections.

 In the present study, molecular biological techniques, that is, PCR-RFLP and sequencing, were applied to bacterial colonies after anaerobic culture, for bacterial identification. The combination of anaerobic culture and molecular biological techniques, utilized also in previous studies [14, 15], seems not only comparatively rapid and low cost but also accurate for the identification of oral microbiota, rather than the mere use of anaerobic culture. However, the number of samples for the analysis in the present study was restricted rather than by only molecular biological technique, for example, PCR or real-time PCR analysis, and the methods did not target noncultivable bacteria such as *Treponema*. In addition, further studies, including examination of biological characteristics of each isolate, are necessary, as the role or complex nature of bacteria in endodontic lesions remains uncertain by the methods in the present study although its characteristics might be speculated based on the general characteristics of reference type strains.

 As a recently developed technique, pyrosequencing has been applied to analyze the composition of the microbiota of infected root canals [18, 19], showing that the microbiota was highly diverse as suggested previously [20] although the technique is high cost for the present. Since the technique is a culture-independent method as well as the real-time PCR amplification and random cloning analysis, it may include not only live but also dead bacterial cells of the microbiota in infected root canals.

 In the preliminary (additional) study, no bacteria were detected in the samples collected just before root canal obturation, that is, on second or third visits during root canal therapy (data not shown), suggesting that adequate treatment may change both the microbiota and the environment of root canals drastically.

 In summary, the microbiota in infected root canals could be rapidly analyzed by the combination of anaerobic culture and molecular biological techniques. The overwhelming majority of the isolates from infected root canals were found to be anaerobic bacteria, suggesting that the environment in root canals is anaerobic and therefore supports the growth of anaerobes.

## Figures and Tables

**Table 1:** Clinical features of subjects and bacterial amounts in the present study.

Case	1	2	3	4	5	6	7	8	9	Mean ± SD
Age	63	63	34	34	34	71	61	59	60	53.2 ± 14.8
Gender	F	M	F	F	F	F	F	F	M	
Tooth^a^	15	31	34	35	17	23	13	32	47	
Size of lesion^b^	4	6	—	—	1	2	3	3	3	

CFU	8.0 × 10^1^	1.0 × 10^4^	3.1 × 10^6^	1.5 × 10^6^	1.4 × 10^5^	7.0 × 10^3^	1.5 × 10^4^	2.7 × 10^2^	9.0 × 10^3^	(0.5 ± 1.1) × 10^6^

^a^A tooth of sampling site is expressed by the FDI two-digit notation.

^b^Size (mm) of lesion of periapical periodontitis estimated by X-ray examination.

**Table 2:** Number of bacterial isolates obtained from infected root canals.

Case	1	2	3	4	5	6	7	8	9	Total
Total isolates	8	31	15	11	13	7	15	9	9	118 (100.0)^a^

Anaerobic	6	30	13	8	13	7	15	6	8	106 (89.8)

* Olsenella*										30 (25.4)
* O. profusa*	2	9	6							
* O. uli*		1				1	8			
* O. *species				3						
* Mogibacterium*										21 (17.7)
* M. timidum*				3	2	6	6		4	
* Pseudoramibacter*										21 (17.7)
* P. alactolyticus*	1	15	1		1			3		
* Propionibacterium*										14 (11.9)
* P. acidifaciens*				2						
* P. acnes*					6					
* P. *species			6							
* Parvimonas*										7 (5.9)
* P. micra*	3				3				1	
* Acidaminococcus *										3 (2.5)
* A. intestini*		3								
* Bifidobacterium*										3 (2.5)
* B. dentium*								3		
* Anaeroglobus*										2 (1.7)
* A. geminatus*									2	
* Dialister*										1 (0.8)
* D. invisus*		1								
* Eubacterium*										1 (0.8)
* E. infirmum*		1								
* Peptostreptococcus*										1 (0.8)
* P. stomatis*									1	
* Shuttleworthia*										1 (0.8)
* S. satelles*					1					
* Slackia*										1 (0.8)
* S. exigua*							1			

Facultatively anaerobic	2	1	2	3	0	0	0	3	1	12 (10.2)

* Streptococcus*										6 (5.1)
* S. anginosus*								2		
* S. gordonii*								1		
* S. intermedius*									1	
* Campylobacter*										3 (2.5)
* C. gracilis*				3						
* Lactobacillus*										3 (2.5)
* L. antri*			2							
* L. crispatus*		1								
* Actinomyces*										2 (1.7)
* A. israelii*	2									

^a^Percentage is given in parenthesis.
